# Rewiring facilitation during inside plastic stent exchange in malignant hilar biliary obstruction

**DOI:** 10.1055/a-2860-0757

**Published:** 2026-05-08

**Authors:** Junichi Kaneko, Kohei Nishizawa, Takuya Otsu, Masaki Takinami, Yurimi Takahashi, Satoshi Tamura

**Affiliations:** 1 Department of Gastroenterology 13773 Iwata City HospitalIwataJapan; 2 Department of Hepatology 13773 Iwata City HospitalIwataJapan

Endoscopic biliary drainage for malignant hilar biliary obstruction (MHBO) often requires multiple stents. Self-expandable metal stent (SEMS) deployment and subsequent replacement could be technically demanding 1 . Suprapapillary placement of inside plastic stents (iPSs) provides an alternative. iPSs might prevent recurrent biliary obstruction (RBO) by reducing duodenobiliary reflux, and they represent removable options in the case of stent dysfunction 2 . In unresectable MHBO, iPSs reportedly allow for time to RBO comparable to that of SEMSs after initial intervention 3 4 . Moreover, scheduled iPS exchange could prevent cholangitis and reduce unplanned hospitalization 5 . However, during multiple iPS exchanges, guidewire access must be re-established in each targeted branch. Rewiring could be challenging in certain branches, e.g., the right posterior sectoral duct (RPSD), leading to a prolonged procedure time or technical failure. Herein, a unique rewiring technique for iPS exchange is described ( Video 1 ).

Guidewire-assisted rewiring during inside plastic stent exchange in malignant hilar biliary obstruction: right posterior sectoral duct rewiring via the stent lumen, followed by stent removal while maintaining guidewire access.Video 1

**Fig. 1:**
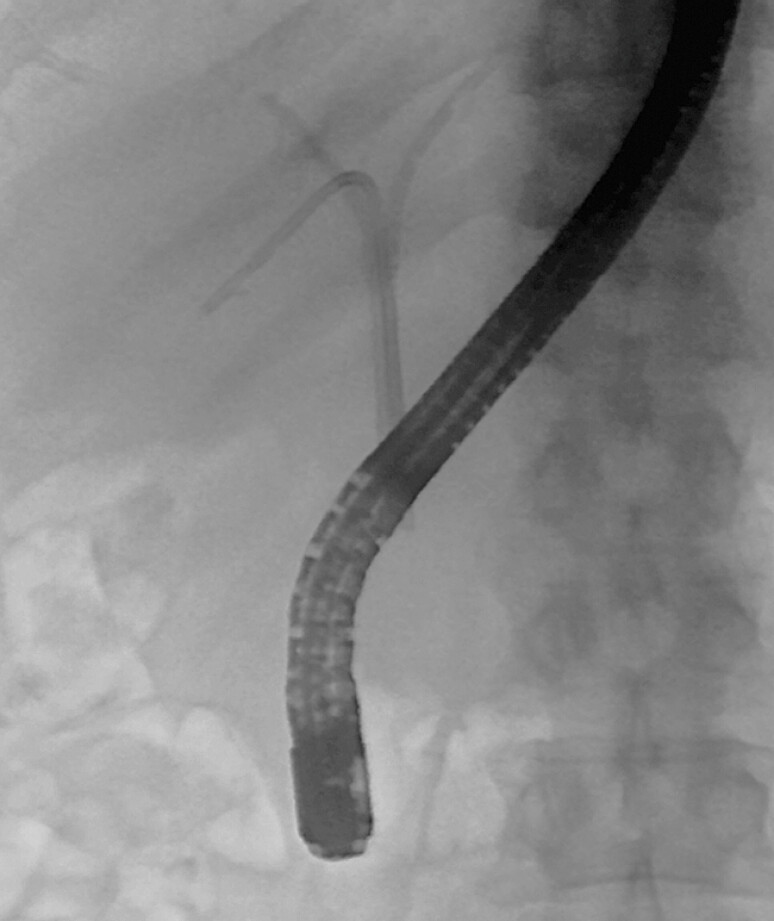
The patient had MHBO of bismuth type IIIa and three suprapapillary inside plastic stents. MHBO, malignant hilar biliary obstruction.

**Fig. 2:**
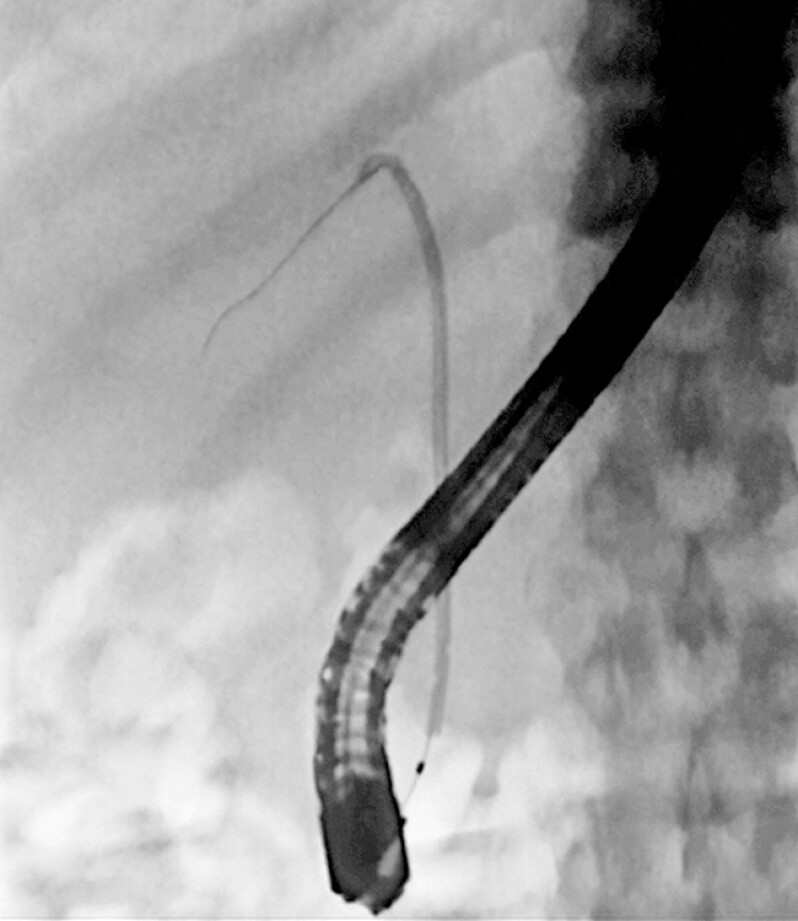
A guidewire was advanced into the right posterior sectoral duct through the plastic stent.

**Fig. 3:**
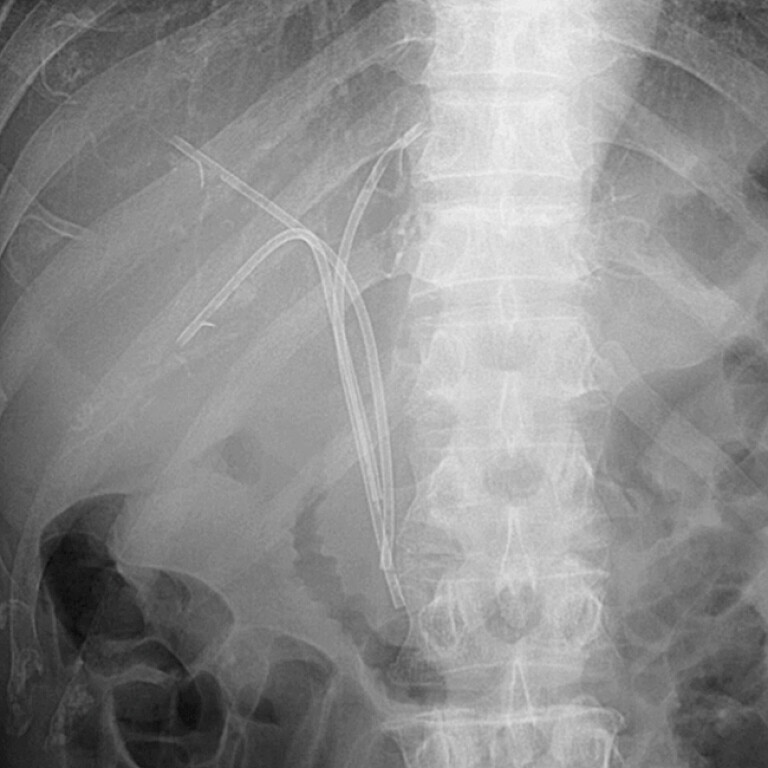
All three inside plastic stents were successfully exchanged.

A man in his 80s with unresectable hilar cholangiocarcinoma presented with RBO-related acute cholangitis 15 months after the initial stent placement. He had MHBO of bismuth type IIIa and three suprapapillary iPSs, i.e., a 7-Fr, 12-cm deep-angle stent in the RPSD, and two 7-Fr, 9-cm light-angle stents, displayed as one in the right anterior sectoral duct (RASD) and another in the left hepatic duct (LHD; Fig. 1 ). First, the iPSs in the RASD and LHD were removed. Next, for the RPSD iPS, the thread was gently pulled using biopsy forceps until the distal end appeared at the major duodenal papilla, with the proximal end seated in the RPSD. A guidewire was advanced through the exposed stent lumen into the RPSD ( Fig. 2 ). The stent was removed while maintaining the guidewire in the RPSD using a snare-over-the-guidewire approach. Finally, guidewire access was established in the RASD and LHD, and all three iPSs were successfully exchanged ( Fig. 3 ).

Endoscopy_UCTN_Code_TTT_1AR_2AZ
